# Cardiac Remodeling and Ventricular Pacing: From Genes to Mechanics

**DOI:** 10.3390/genes15060671

**Published:** 2024-05-23

**Authors:** Onoufrios Malikides, Emmanouel Simantirakis, Evangelos Zacharis, Konstantinos Fragkiadakis, George Kochiadakis, Maria Marketou

**Affiliations:** 1 Department of Cardiology, University General Hospital of Heraklion, 71003 Heraklion, Greece; simantir@uoc.gr (E.S.); ezacharis@yahoo.gr (E.Z.); fragkiadakisk@hotmail.com (K.F.); kochiadg@gmail.com (G.K.); maryemarke@uoc.gr (M.M.); 2 Medical School, University of Crete, 71003 Heraklion, Greece

**Keywords:** cardiac remodeling, pacing

## Abstract

Cardiac remodeling and ventricular pacing represent intertwined phenomena with profound implications for cardiovascular health and therapeutic interventions. This review explores the intricate relationship between cardiac remodeling and ventricular pacing, spanning from the molecular underpinnings to biomechanical alterations. Beginning with an examination of genetic predispositions and cellular signaling pathways, we delve into the mechanisms driving myocardial structural changes and electrical remodeling in response to pacing stimuli. Insights into the dynamic interplay between pacing strategies and adaptive or maladaptive remodeling processes are synthesized, shedding light on the clinical implications for patients with various cardiovascular pathologies. By bridging the gap between basic science discoveries and clinical translation, this review aims to provide a comprehensive understanding of cardiac remodeling in the context of ventricular pacing, paving the way for future advancements in cardiovascular care.

## 1. Introduction

Pacemaker technology has rapidly evolved from the initial external electronic cardiac device in the late 1940s with a restricted lifespan, transitioning from conventional transvenous pacemakers to leadless devices. The initial use of permanent artificial pacemakers (PPMs) was primarily aimed at treating heart blocks and frequent episodes of Adams–Stokes syndrome or bradyarrhythmic cardiac arrest [1,2]. Nowadays, the implementation of PPMs has dramatically increased and is tailored to address a wide range of bradyarrhythmias [3]. The annual count of PPM placements continues to rise steadily, surpassing a global rate of over 1 million new implants per year [4]. 

The implantation of PPMs notably enhances the quality of life for patients; however, right ventricular apical pacing (RVAP) results in the asynchronous contraction of the heart chambers that can potentially trigger cardiac remodeling with functional and structural abnormalities [5,6]. Multiple studies have demonstrated that prolonged RVAP in individuals with preserved left ventricular (LV) function and no prior history of heart failure or coronary artery disease can lead to ventricular dyssynchrony, cardiac remodeling, and ultimately, the deterioration of systolic function over time and heart failure (HF) [7,8]. 

The interaction between cardiac remodeling and ventricular pacing entails intricate processes, such as mechanical stress, gene expression, neurohomonal activation, electrical dyssynchrony, as well as apoptosis, and cell death [9]. Initially, these changes represent an adaptive process of the heart to preserve cardiac function under conditions of abnormal LV excitation [10]. However, they result in heart dysfunction, cardiac output worsening, and unfavorable cardiovascular outcomes such as the development of ventricular pacing-induced cardiomyopathy [11,12,13]. Pathological molecular mechanisms and complex signaling pathways are involved in these myocardial changes and regulate the prevailing phenotypes. 

A better understanding of the mechanisms underlying the relationship between artificial cardiac pacing and cardiac remodeling is essential for developing strategies to mitigate its detrimental effects.

**Figure 1:**
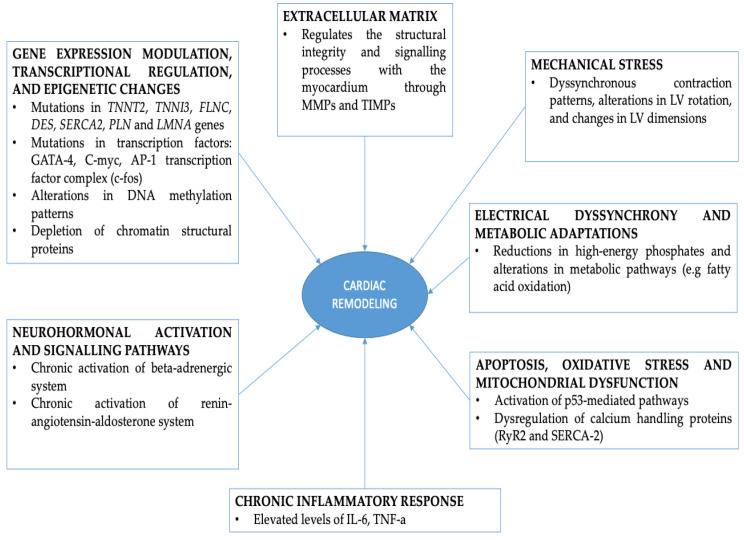
Major pathophysiological pathways involved in cardiac remodeling in patients with permanent right ventricular apical pacing.

The purpose of this review is to provide a comprehensive insight into the correlation between ventricular pacing and cardiac remodeling by exploring underlying pathophysiological mechanisms spanning from genetic factors to heart function and mechanics. By elucidating these mechanisms, this review aims to shed light on potential future therapeutic targets for preventing and/or delaying the onset of cardiac abnormalities induced by chronic artificial pacing. Figure 1 depicts a summary of the major pathways involved in cardiac remodeling.

## 2. Transcriptional Regulation and Gene Expression Modulation during RVAP

Increasing evidence has indicated that gene expression, leading to cardiac remodeling, is mediated by transcription factors and the epigenetics process [14,15,16] and occurs as a result of dysregulation in genes encoding a variety of proteins, including sarcomeric and structural proteins. Gene expression dysregulation may control excitation–contraction coupling, as well as the production of contractile proteins [17]. Altered energetic and metabolic processes of the myocyte can alter gene expressions that regulate calcium handling or cytoskeleton integrity and may also lead to adverse cardiac remodeling. Moreover, independent genetic variants have been described in the literature and linked with cardiac remodeling affecting various aspects of cardiac structure and function, such as myocardial hypertrophy, fibrosis, contractility, and electrical signaling [18]. Predisposing mutations in genes such as *ACTC* (cardiac actin gene), *TNNT2* (encoding human cardiac troponin T2), and *TNNI3* (encoding cardiac troponin I3), as well as in sarcomeric proteins like filamin C—an actin-binding protein (encoded by the *FLNC* gene)—are linked to cardiac remodeling and the development of severe cardiomyopathies [18,19,20,21]. Mutations in the *DES* gene—encoding the intermediate filament protein desmin, which serves as a mitochondrial anchor—were found to cause cardiac remodeling and, in consequence, severe cardiomyopathies, frequently associated with ventricular or atrial arrhythmias. For instance, *DES-c.735G>C* leads to abnormal desmin filament connections associated with restrictive caridiomyopathy and atrial fibrillation [22]. A novel heterozygous *R406W* de novo mutation in *DES* was detected in a patient presenting with cardiomyopathy alongside atrioventricular block [23]. This observation suggests that the presence of this mutated protein is linked to pathological cellular changes and remodeling [23]. Individuals with cardiomyopathies may carry pathogenic variants in the *LMNA* gene, which encodes the nuclear envelope proteins lamin A and C; these proteins are crucial for regulating mechanotransduction within cardiac cells [24]. Epigenetic changes, including alterations in gene expression, seem to be involved in the pathophysiology of RVAP-induced cardiac remodeling [16]. In a recent study, it was presented that the overall methylation of *CpG* sites was higher in individuals with dilated cardiomyopathy compared to healthy controls [25]. Distinct DNA methylation patterns were also observed in left ventricular tissues from individuals with dilated cardiomyopathy, affecting genes such as *LY75* (lymphocyte antigen 75), *ERBB3* (tyrosine kinase-type cell surface receptor HER3), *HOXB13* (Homeobox B13), and *ADORA2A* (adenosine receptor A2A) [26]. Additionally, in individuals with HF, the depletion of the *CTCF* gene, which encodes transcriptional repressor *CTCF*, a ubiquitous chromatin structural protein, suggests the potential role of chromatin remodeling complexes in cardiac remodeling and cardiomyopathies [27].

Pathological and increased LV excitation seem to affect ATPase sarcoplasmic/endoplasmic reticulum Ca2+ transporting 2 gene (SERCA2) expression, which regulates cardiac contractility [28]. A previous study indicated that the duration of paced QRS complexes is correlated with changes in *SERCA2* expression, which precede the decline in LV function and adverse remodeling in patients undergoing permanent pacemaker implantation [28]. Specifically, as paced QRS duration increases, there is a significant reduction in *SERCA2* expression, which serves as an indicator of LV function. Notably, patients with narrow-paced QRS complexes do not demonstrate statistically significant changes in *SERCA2* expression [29]. According to the published literature, *SERCA2* is considered both a marker and a therapeutic target for HF [30,31]. Indeed, *SERCA2* levels decline with HF progression, but they increase upon remission of the condition [32,33]. Phospholamban (encoded by the *PLN* gene) is another key protein involved in cardiomyocyte calcium handling, acting as a primary regulator of SERCA2. When unphosphorylated, PLN reduces SERCA’s affinity for Ca2+, inhibiting calcium uptake. Phosphorylation at serine 16 by PKA or threonine 17 by Ca^2+^/calmodulin-dependent protein kinase II (CaMKII)—a serine/threonine-specific protein kinase that is regulated by the Ca^2+^/calmodulin complex—reverses phospholamban-mediated inhibition, boosting SERCA2 activity and calcium uptake [34]. Several variants in the PLN gene have been described in HF, such as the *c.40_42delAGA* pathogenic variant; a heterozygous deletion of arginine 14 (Arg14del) in the PLN protein has been associated with a high risk of developing malignant ventricular arrhythmias and HF, often diagnosed as dilated cardiomyopathy or arrhythmogenic right ventricular cardiomyopathy [35]. For instance, a study examining the hearts of mice homozygous for the *PLN-R14del* pathogenic variant observed the upregulation of fetal genes and extensive myocardial fibrosis, leading to cardiac remodeling compared to wild-type mice [36]. In a recent study by Simantirakis et al., *SERCA2*, *MYH* genes (encoding α- and β- myosin heavy chains (MHC), proteins that regulate myocardial contractile function and hypertrophy were observed to alter after examining peripheral blood mRNA levels in patients with long-term RVAP. *SERCA2* and *MYH6* gene expressions were decreased in comparison to *MYH7*, which was increased until 6 months follow-up [28]. In a cohort of 52 consecutive patients who underwent pacemaker implantation for bradycardic indications, 25% of patients demonstrated significant LV remodeling. After 4 years, the LV end-systolic and end-diastolic diameters were significantly increased, and EF declined [29]. Early alterations in gene expression were associated with a deterioration in LV function and geometry that became apparent months later [29]. Similarly, a prospective randomized controlled study found that individuals on chronic high-dose RVAP exhibited higher expression levels of *OPA1* (OPA1 mitochondrial dynamin-like GTPase) and *SERCA2* genes compared to those with right ventricular outflow tract (RVOT) pacing. These early mRNA changes were linked to the subsequent deterioration in the global longitudinal strain (GLS) observed during echocardiography one month following pacemaker implantation [37]. The *OPA1* gene encodes a protein that localizes to the inner mitochondrial membrane, regulates several important cellular processes, including the stability of the mitochondrial network, and plays a role in the maintenance of the DNA within mitochondria.

Experimental data have shown that the heart undergoes a significant shift from its usual gene program to one reminiscent of fetal development when encountering hemodynamic or metabolic stressors [38]. Beyond the *SERCA2* gene, additional gene alterations include the expression of proteins such as α- and β-MHC, encoding the atrial natriuretic peptide (ANP), a transcription factor *c-myc*, a component of the AP-1 transcription factor complex *c-fos*, transforming growth factor β (TGFB), as well as the transcription regulator GATA binding protein 4 (GATA-4) [38]. Moreover, an additional potential pathophysiological mechanism contributing to heart failure in individuals with right ventricular pacing is the acceleration of the apoptotic process. Experimental animal studies of right ventricular pacing suggest the heightened expression of apoptotic agonists such as TP53 (tumor protein p53) [39], alongside the reduced expression of inhibitors like BCL2 (BCL2 apoptosis regulator) [40].

Another proposed pathophysiological mechanism suggests that during stress conditions (pressure overload, ischemia, unloading), the heart alters its metabolism using less commonly used pathways, such as the hexosamine biosynthetic pathway. This leads to the accumulation of metabolic signals, mainly from glucose metabolism, which subsequently influences gene expression [41]. The main features include the dysregulation of *MYH6* and *MYH7* and the alteration of cardiac metabolism from oxidizing fatty acids to utilizing carbohydrates for energy provision. Additional changes have been detected in the genes *c-myc* and *c-fos*, which are involved in adverse cardiac remodeling [38]. In canine models of pacing-induced cardiomyopathy, further restructuring and adaptations in the sarcomeres have been observed. Unlike the control group, animals with pacing-induced cardiomyopathy exhibited a decrease in the expression of the N2BA titin isoform (characterized by a longer spring) and a greater prominence of the N2B isoform (known for its shorter spring). Interestingly, pacing did not affect the overall quantity of titin but rather influenced the expression levels of the stiffer N2B isoform. Consequently, pacing leads to alterations in diastolic dysfunction in heart failure by reshaping the muscle structure [42]. A recent animal study investigating LV remodeling during the progression of heart failure induced by rapid ventricular pacing found that *NPPA* gene expression (encoding ANP) in those with ventricular pacing was dramatically increased in the overt chronic HF characterized by an increase in LV systolic wall stress [7]. It is noteworthy that the *NPPA* gene was not observed to increase during the asymptomatic phase of left ventricular dysfunction. This suggests that left ventricular *NPPA* gene expression is strongly activated during the later changes in heart failure, independent of further increases in wall stress [7].

Over the past decade, genetic-based investigations in mice have identified a specific set of transcriptional regulators that directly influence various aspects of cardiac cell lineage commitment and/or heart morphogenesis. It is noteworthy that in response to specific conditions like mechanical stress, these transcription factors are reactivated, contributing to the progression of cardiac remodeling by promoting hypertrophic enlargement and/or dilated cardiomyopathy [17,43]. Typically, these transcription factors are activated through signal transduction pathways initiated by membrane-bound receptors in response to neural–humoral agonists [44]. GATA-4, a transcriptional factor, was found to alter the cardiac hypertrophic response or the survival of myocytes through the regulation of numerous cardiac genes, including *NPPA*, *NPPB*, *MYH6*, and *MYH7.* This is mediated when GATA-4 undergoes enhancement via phosphorylation in response to various stimuli implicated in heart failure and/or cardiac hypertrophy, such as pressure overload, endothelin-1, and angiotensin II [45].

Additionally, in HF patients, a positive response to cardiac resynchronization therapy (CRT) was associated with favorable changes in genes regulating LV contractile function and hypertrophy [33]. In a study on adult dogs, CRT was found to improve gene dysfunction primarily in the anterior LV wall. CRT reversed the changes in gene expression in the anterior wall, indicating the widespread impact of biventricular pacing on the transcriptome of the ventricles, reaching beyond the specific pacing location in the lateral wall [46]. An overview of the most important studies regarding gene expression and cardiac remodeling is presented in Table 1.

## 3. Molecular Pathways Involved in Cardiac Remodeling during RVAP

Molecular pathways involved in ventricular pacing-induced cardiac remodeling include neurohormonal activation, apoptosis, inflammatory response, calcium handling abnormalities, oxidative stress, extracellular matrix remodeling, and mitochondrial dysfunction.

### 3.1. Neurohormonal Activation and Dignaling Pathways

The signaling pathways of neurohormonal systems (such as β adrenergic and the renin–angiotensin–aldosterone system (RAAS) pathways) play a significant role in maladaptive remodeling processes and arrhythmogenicity. In heart failure, the chronic activation of the β-adrenergic pathway can lead to the downregulation and desensitization of β-adrenergic receptors (β-ARs), resulting in impaired contractile function and responsiveness to sympathetic stimulation [48]. In addition to changes in the genetic background, neurohormonal pathways play an important role, such as the β-ARs and the long-term effect of the sympathetic nervous system, as well as the RAAS. In fact, it seems that treatment with β-blockers and RAAS inhibitors reduces the risk for pacemaker-induced cardiomyopathy (PiCM) [10]. In a small study including eight patients with normal LV function, temporary ventricular pacing wires were used to examine both the neurohormonal response and cardiac mechanical alterations [49]. The authors concluded that individuals subjected to RVAP exhibited an increase in cardiac norepinephrine levels [49]. This was accompanied by a decrease in LV chamber efficiency, slowing the rate of LV isovolumic relaxation and elevating LV end-diastolic pressure [49]. RVAP can stimulate one or more afferent reflex pathways that lead to reduced LV contractility, the decreased firing rate of ventricular mechanoreceptors, and finally, the withdrawal from the vagal tone. These processes collectively result in increased sympathetic excitation, contributing to heightened cardiac sympathetic drive [50]. Hamdan et al. investigated sympathetic activity in individuals with ventricular pacing and a reduced ejection fraction and found that sympathetic nerve activity was greater in RVAP compared to left or biventricular pacing [50]. There are two potential mechanisms that could explain the decreased sympathetic activity observed. Firstly, it might be mediated through arterial baroreflex-mediated sympathoexcitation, given that the reduction in sympathetic nerve activity coincided with an increase in arterial blood pressure. Secondly, pacing on the cardiopulmonary baroreceptors could lead to increased cardio inhibition, resulting in a decrease in peripheral sympathetic activity [51,52]. Luchner et al. reported that HF induced by rapid ventricular pacing results in elevated levels of angiotensin II during the advanced stages of congestive HF [53]. Angiotensin II is central to cardiac remodeling, impacting both structural and electrical aspects. It induces cardiac myocyte hypertrophy and interstitial fibrotic changes in cardiac fibroblasts, contributing to structural remodeling. Additionally, it fosters electrical remodeling by promoting oxidative stress and inflammation, thus exerting proarrhythmic effects [54]. According to Agha et al., in individuals with a high burden of RVAP, the administration of angiotensin-converting enzyme inhibitors/angiotensin receptor blockers and β-blockers seems to mitigate the risk of pacing-induced cardiomyopathy [55].

### 3.2. Apoptosis, Oxidative Stress, Calcium Handling Abnormalities and Mitochondrial Dysfunction

Paced hearts typically exhibit minimal cardiac hypertrophy but show ongoing ventricular remodeling, along with indications of myocyte loss primarily linked to apoptosis, as well as extensive alterations in the extracellular matrix [56]. The precise mechanisms underlying the initiation and progression of the apoptosis process remain incompletely understood. Several factors are established causing the induction of apoptosis in cardiomyocytes during heart failure, including disruption to Ca2+ cycling, leading to increased intracellular Ca2+, mechanical stress, or the production of superoxide anions resulting from mitochondrial damage [57]. Although the apoptotic cascade was not completed, failing myocardium led to disrupted cellular processes (without rupturing the sarcolemma) and contributed to the extensive mitochondriac loss of cytochrome c and, finally, to heart dysfunction [58]. In the pacing model of HF in dogs, it was observed that the reduced wall thickness and the increase in LV dimensions were associated with a reduction in cell number. In addition, in the group with pacing-induced HF, several proteins involved in the cascade of apoptosis (Fas, Fas-L, caspases-2, and -3) were found to be elevated, and inhibitors of apoptosis, such as Bcl-2, were down-regulated [40]. The transcriptional regulators p-53 of Bcl2- and Bax, as well as p53-dependent genes, were also found to be activated in right ventricular pacing dogs, indicating their critical role in the modulation of myocyte apoptosis in pacing-induced heart failure [39]. While P53 is recognized for its role in mediating cardiomyocyte apoptosis triggered by stimuli-like hypoxia and oxidative stress, it also serves pivotal functions beyond transcriptional regulation. Firstly, P53 stimulates nuclear and mitochondrial DNA base excision repair and interacts directly with mitochondrial membranes to facilitate cytochrome c release. Secondly, it involves DNA repair mechanisms and apoptosis. Therefore, the significant increase in P53 and P21 expression observed in paced hearts suggests the activation of a DNA damage response [47]. In another study examining the subcellular mechanism underlying rapid pacing-included cell death, it was observed that a calcium/calmodulin-dependent protein kinase II (CaMKII) and reactive oxygen species (ROS) production were involved in RP-induced apoptosis. CAMKII modifications of the cardiac ryanodine receptor-2 (RYR2) lead to enhanced diastolic Ca2+ release and mitochondrial Ca2+ overload, resulting in cell death [59]. Similarly, another study in animals showed that ventricular pacing, compared with atrial pacing, led to profound LV alterations and mechanical dyssynchrony in both apoptotic pathways and calcium handling in the early stages of pacing-induced cardiomyopathy. The animals with ventricular pacing had abnormal LV sarcoplasmic reticulum calcium uptake and LV calcium-handling protein expression, such as a reduction in RYR2, increase in Na+/Ca2+ exchanger, or decline in sarcoplasmic reticulum Ca2+ ATPase [60]. Additionally, in pacing-induced HF canines, a higher occurrence of large-scale mitochondrial deletions was observed in myocardial tissue compared to control animals, suggesting the significant role of mitochondrial dysfunction in the development of cardiomyopathy [61].

### 3.3. Extracellular Matrix Remodeling and Inflammatory Response during RVAP

RVAP affects the extracellular milieu by modulating the function of various metalloproteinases and collagen [62]. The extracellular matrix (ECM) of the ventricular myocardium is a dynamic tissue that contains an organized structural network of matrix proteins, including metalloproteinases (MMPs), tissue inhibitors of metalloproteinases (TIMPs), and fibrillar collagens [62]. TIMPs have a pivotal role in the process of remodeling the ECM through the degradation of components of the matrix, such as collagen and elastin. Furthermore, ECM is richly invested with a diversity of signaling molecules, including angiotensin, endothelins, and cytokines. According to the literature alterations in the functional expression of TIMPs and MMPs were identified as the cause of structural remodeling in heart failure [63]. The expression of matrix MMPs and TIMPs, as well as collagen types I and II, were increased in animal models with RVAP, and the expression of type II collagen in the lateral wall [50]. Additionally, there was activation and protein expression of MMP-2 and MMP-9, along with the protein expression of TIMP-1 and TIMP-3 in the lateral wall [64]. The mechanism underlying the activation of MMPs and TIMPs in right ventricular pacing-induced fibrosis is not fully understood. According to this study, mechanical stress could be a contributing factor, as the effects of fibrosis and structural alterations were mostly pronounced in regions of the ventricle experiencing late activation and higher stress [65]. Another study proposed a potential mediation by angiotensin II. Experimental studies have shown that sympathetic nerve stimulation induced by angiotensin II may explain a mechanistic pathway leading to MMP activation [66]. Remodeling in atrial cardiomyopathy led to distinct alterations in MMP activity and compensatory adjustments in phospholamban levels. This has been evidenced by a study investigating left atrial myocardium from dogs with rapidly induced atrial failure. While SERCA2 levels remained unchanged compared to healthy controls, there was a significant decrease in phospholamban levels observed in the atrial samples from rapidly paced dogs [67].

Chronic ventricular pacing can induce persistent mechanical stress and hemodynamic changes, which, in turn, stimulate an inflammatory response within the myocardium, including the expression of cytokines. This inflammation is a contributing factor not only to myocardial remodeling but also to an elevated risk of arrhythmias and worsening HF. Additionally, there is evidence suggesting that right ventricular pacing facilitates the inflammatory response within the myocardium, mediated by inflammatory cytokines, such as IL-6 [68] and TNF-α [69]. Among the cytokines implicated in pacing-induced inflammation, IL-6 has been found to be elevated in the plasma of individuals undergoing RVAP. Moreover, in patients with heart failure, there is a significant correlation between plasma IL-6 concentration and plasma norepinephrine levels, suggesting a potential link between neurohormonal activation and cytokine-mediated inflammation in the context of ventricular pacing-induced HF [68].

The overexpression of TNF-α in patients with HF has been implicated as a potential cause of LV dysfunction through activation of the pro-apoptotic effect, contributing to cardiomyocyte death as well as promoting the expression of matrix MMPs. While some studies have suggested that TNF-α expression may be elevated in patients undergoing right ventricular pacing, the exact role and significance of TNF-α in pacing-induced cardiac remodeling are not fully understood [60,69]. In early studies of permanent pacing, inflammation was noted to arise primarily as a result of electrical stimulation, followed by the secondary observation of inflammation localized at the pacing site [70]. In an animal model, the induction of transmural dyskinesia by suprathreshold epicardial LV activation was associated with a localized inflammatory response in the epicardium. Additionally, researchers observed a sequential process where there was a loss of endocardial thickening, followed by a decline in endocardial function [71]. The cellular and extracellular remodeling induced by RVAP is of notable significance. The dyssynchrony associated with this pacing method appears to induce alterations in intracellular metabolism, particularly within the mitochondrial respiratory chain, which substantially affects the structural status and the functional performance of the paced heart.

### 3.4. Electical Dyssynchrony, Metabolic Adaptations and Energetics

Electrical dyssynchrony is associated with alterations in the electrophysiology, structure, and metabolism of the heart. These alterations represent three mechanisms of compensation aimed at delaying or mitigating the progression of HF. However, when these mechanisms become maladaptive, they contribute to the progression of the HF phenotype [72]. A canine model with tachypacing-induced HF has shown that electromechanical dyssynchrony is associated with metabolic remodeling. Biopsies obtained from the LV apex were analyzed for metabolic pathways, indicating notable differences between those with synchronous HF and those with dyssynchronous HF. Compared to synchronous HF, individuals with dyssynchronous HF exhibited reduced levels of ATP, phosphocreatine, and creatine and a lower phosphocreatine/ATP ratio. Moreover, myocardial levels of carnitine (a mitochondrial fatty acid carrier) and fatty acids were significantly diminished in dyssynchronous HF compared to synchronous HF. Although both synchronous and dyssynchronous HF showed reductions in creatine and intermediates of glycolysis and the tricarboxylic acid (TCA) cycle, the degree of reduction varied between these two conditions. These findings suggest a systemic mismatch between substrate supply and demand in pacing-induced heart failure. The energy deficit observed in diastolic HF, but not in systolic HF, could be explained by a significant reduction in the delivery of fatty acids to the ß-oxidation pathway, primarily due to a reduction in the myocardial carnitine content [73]. Similarly, in atrial tissues from paced animals, an investigator found severely reduced activities of the phosphotransferase enzymes, creatine kinase, and adenyate kinase, as well as the depletion of high-energy phosphates (ATP and creatine phosphate), indicating that atrial bioenergetics are also affected in the failing paced heart [74]. Reduced levels of myocardial respiratory complex I [75], III, and V activities and mitochondrial fatty acid b oxidation have been also observed in the pacing-induced HF model obtained from LV tissues [61,76].

## 4. Mechanical Stress and Structural Remodeling

Ventricular pacing has significantly improved patients’ quality of life and increased survival rates. However, the remodeling caused in the cardiac tissue by the abnormal conduction in the electrical stimulus in the myocardium is currently a field of special research interest. Several studies have shown that prolonged paced QRS duration is linked to adverse LV remodeling and indicates the onset of pacing-induced cardiomyopathy. RVAP leads to mechanical desynchronization, diminished myocardial perfusion, and decreased left ventricular EF [77]. Persistent ventricular dyssynchrony induced by prolonged endovenous RVAP results in detrimental consequences, such as LV remodeling, dilation, asymmetrical hypertrophy, and decreased exercise tolerance (Table 2). Microscopic findings include the diffuse fibrosis of ECV [78], the loss of the t-system, the separation of RYR2 from sarcolemma structures [79], and low PLN expression. This pacing method involves electrical propagation through myocardial tissue rather than the natural His–Purkinje conduction system, leading to slower electrical propagation and an activation pattern comparable to the left bundle branch block (LBBB) [80].

Henceforth, investigating cardiac tissue remodeling arising from right ventricular pacing presents a multifaceted challenge, encompassing mechanical, genetic, cellular, and neurohormonal dimensions. The primary objective is to identify therapeutic approaches aimed at averting or delaying progression towards HF.

Prolonged RVAP induces the eccentric activation of the ventricles and, in most cases, dyssycnhronous contraction. This can lead to enduring structural alterations in the myocardium, which may contribute to unfavorable LV remodeling. Further alterations include left atrial remodeling, functional mitral regurgitation, and abnormalities related to myocardial coronary perfusion [81]. While the precise mechanisms by which the mechanical load is transmitted to myocardial cells remain unclear [82], it is widely recognized that the stretch serves as a significant trigger for altering cellular and organ function, both in vitro and in vivo [83]. In cases of LBBB, the initial systolic prestretch and increased external work in regions activated later in the cardiac cycle provide plausible explanations for the often more pronounced remodeling processes observed in these regions [84]. Apical pacing leads to a reduced rate of change in LV pressure and induces a dyssynchronous pattern of ventricular contraction. This dyssynchrony results in an asynchronous myocardial contraction, leading to reduced stroke volume. Furthermore, the mismatch in relaxation between early and late-contracting myocardial areas leads to a decrease in LV filling duration [85]. In addition, in a study by Matsuoka et al., using ultrasound imaging, it was found that RVAP reduces both apical and basal LV rotation, leading to a delay in LV apical-basal rotation and an impaired LV twist [86]. However, it is anticipated that similar pathophysiological mechanisms could apply to patients with electrical dyssynchrony due to pacing. The normal contraction of the LV is coordinated in a way that ensures synchronous fiber shortening throughout the chamber, facilitated by the Purkinje conduction system. This coordination can be disrupted by conditions like LBBB or pacing. In such cases, the left ventricle’s contraction becomes inefficient, causing one side to move inward before the other, leading to a back-and-forth shifting of blood volume within the chamber instead of the effective ejection, ultimately resulting in decreased systolic performance [23,87]. A cohort study using cardiac magnetic resonance imaging (cMRI) in order to investigate 62 patients with or without prolonged left ventricular dyssynchrony observed that those with progressive left ventricular dilatation (PLVD) were associated with a higher volume of focal (late gadolinium enhancement [LGE]) and diffuse fibrosis (extracellular volume [ECV] [88]).

Whether dyssynchrony independently contributes to myocardial structural remodeling or is in combination with other factors remains unknown. However, another pathophysiological mechanism that can explain the association is that dyssynchrony provides mechanical stress primarily in early systole and late systolic contractions against an increased afterload. A recent study in the canine model of dyssynchronous heart failure indicated changes in the transverse tubular system (t-system) and the arrangement of ryanodine receptor (RyR) clusters. Unlike the synchronous HF model induced by atrial tachypacing, where such remodeling was absent, HFpEF exhibited a spectrum of remodeling, ranging from minor modifications in certain left ventricular myocytes to almost the complete loss of the t-system and separation of RyRs from sarcolemmal structures in lateral cells [89].

These cascading events ultimately culminate in the activation of myocardial tissue fibrosis, leading to the subsequent development of heart failure. More specifically, contraction dyssynchrony adversely impacts the mechanical performance of the myocardium. Specifically, the asynchronous contraction of different segments of the myocardial wall results in reduced preload and contractility. Consequently, this leads to diminished stroke volume and cardiac output [90].

The incidence of pacemaker-induced cardiomyopathy has risen in tandem with the rapid increase in the number of pacemaker implantations, significantly impacting both the quality of life and survival expectancy of affected patients. Key areas of research interest include identifying risk factors, namely which patients are more susceptible to pacemaker-induced cardiomyopathy development, as well as exploring therapeutic interventions to mitigate its effects. Some of the studied risk factors for pacemaker-induced cardiomyopathy are the presence of reduced LVEF before PPM implantation, the presence of asynchronous ventricular contraction (wide QRS), and the degree of right ventricular pacing. Data in the existing literature regarding the degree of pacing and PiCM development are equivocal. Three main studies found different percentages of right ventricular pacing as predictors of pacemaker-induced cardiomyopathy development: >20% [89], >40% [91], and >60% [92] for the burden of right ventricular pacing and interventricular dyssynchrony.

## 5. Conclusions

In conclusion, this review underscores the intricate interplay between cardiac remodeling and ventricular pacing, illuminating the field from molecular mechanisms to clinical implications. Through the lens of genetics, cellular signaling pathways, and biomechanical alterations, we traversed the landscape of cardiac remodeling, elucidating its profound impact on ventricular function and pacing strategies. From the early stages of arrhythmogenic substrate formation to the adaptive responses in myocardial structure and function, our study underscored the dynamic nature of cardiac remodeling in the context of ventricular pacing. Insights gleaned from this synthesis not only deepen our understanding of the underlying pathophysiology but also pave the way for refined therapeutic interventions aimed at optimizing pacing strategies and improving patient outcomes. As we navigate the complexities of cardiac remodeling and ventricular pacing, it is imperative to continue bridging the gap between basic science discoveries and clinical translation, fostering a holistic approach toward enhancing cardiac health and advancing patient care.

## 6. Future Directions

Looking ahead, future research endeavors in the realm of cardiac remodeling and ventricular pacing are poised to unlock novel insights and propel clinical innovation. Firstly, exploring the burgeoning field of precision medicine holds promise in tailoring pacing strategies to individual patient characteristics, encompassing genetic predispositions, phenotypic profiles, and dynamic changes in cardiac structure and function. The integration of advanced imaging modalities, such as cardiac magnetic resonance imaging and computational modeling techniques, could offer a refined understanding of ventricular mechanics and guide personalized therapeutic decision making. Additionally, elucidating the role of emerging technologies, including leadless pacing systems and multisite pacing configurations, may offer enhanced efficacy and minimize adverse remodeling effects. Moreover, leveraging the power of big data analytics and artificial intelligence holds the potential to identify novel biomarkers for the early detection of adverse remodeling and predicting patient-specific responses to pacing interventions. By embracing these multidisciplinary approaches and fostering collaborative endeavors between basic scientists, clinicians, and engineers, the future landscape of cardiac remodeling and ventricular pacing holds promise for transformative advancements in cardiovascular care.

## Figures and Tables

**Table 1:** Studies showing alterations in gene expression levels following permanent artificial pacing.

	Study Group	Gene Expression	Follow-Up	Findings
Luchner et al. [7]	Eleven dogs underwent implantation of a programmable cardiac pacemaker for rapid ventricular pacing.	*NPPA* gene expression levels were evaluated in myocardial tissue samples.	38 days	*NPPA* gene levels were elevated in all samples from overt CHF ventricular tissue samples compared to being barely detectable in control and asymptomatic left ventricular dysfunction dogs. A positive signal for *NPPA* mRNA was present in all left atrial samples from the control, asymptomatic left ventricular dysfunction, and overt CHF animals. Left atrial ANP concentrations exceeded left ventricular ANP and showed a tendency to increase during overt CHF.
Arkolaki et al. [28]	52 patients with bradycardic conditions (group A-24 individuals AVCD and group B-28 patients with sinus node disease).	*SERCA2*, *MYH6*, and *MYH7* gene expression levels were evaluated in peripheral blood.	12 months	In group A, *SERCA2* and *MYH6* genes decreased compared to *MYH7* which increased. In total, 25% of patients from group A demonstrated significant LV remodeling. Early alterations in gene expression were associated with a deterioration in LV function and geometry that became apparent months later on echocardiographic evaluation.
Vanderheyden et al. [33]	Twenty-four patients were referred for CRT implantation.	*MYH6*, *MYH7*, and *NPPB* gene expression levels were evaluated in the myocardial tissue sample.	4 months	Responders to CRT were associated with an increase in MYH6 mRNA levels and the ratio of α-/β-MHC proteins. Furthermore, a decrease in *NPPB* mRNA levels was observed in the same population.
Eijgenraam et al. [36]	6 wild-type mice, 10 heterozygous (PLNR14^Δ/+^) mice and 13 homozygous (PLN-R14 ^Δ/Δ^) mice	Homozygous mice showed upregulation of fetal genes as follows: elevated expression of the *NPPA* and *NPPB* gene and increase in the ratio of *MYH7/MYH6* gene expression	20 months	Mice *PLN-R14* ^Δ/Δ^ demonstrated increased LV end-diastolic and end-systolic volumes, decreased stroke volume and ejection fraction, as well as significantly decreased survival compared to wild-type mice Elevated LV expression of fibrotic genes was observed in homozygous mice Heterozygous mice developed cardiomyopathy at a later age; cardiac sections showed PLN protein aggregation in some of the cardiomyocytes and an increase in myocardial fibrosis
Xu et al. [37]	60 patients with complete AV block and preserved LVEF (≥50%) (group A-30 RVA pacing, group B-RVOT pacing).	*OPA1* and *SERCA2a* gene expression levels were evaluated in peripheral blood.	24 months	*OPA1* and *SERCA2a* mRNA levels were significantly lower in group A compared to group B. Compared with the baseline value, the mRNA levels of *SERCA2a* and *OPA1* were decreased in group A in the first month of evaluation. In group B, this difference was not statistically significant between the initial and final mRNA levels of both genes.
Wu et al. [42]	Six tachycardia-induced dilated cardiomyopathy canine models compared to 14 controls.	N2B titin isoform (short spring), N2BA titin isoform (long spring), and obscurin- 800 kDa elastic protein were involved in signaling processes in sarcomeric restructuring and were evaluated in myocardial tissue samples.	4 weeks	In paced animals, in contrast to the control, the expression of N2BA titin was reduced and the N2B expression was more prominent. Pacing did not influence the total amount of titin, but the expression level of the stiffer N2B isoform was influenced. Obscurin was upregulated by pacing.
Marin-Garcia et al. [47]	Eleven dogs underwent continuous rapid right ventricular pacing compared to ten normal dogs who served as controls.	Large-scale mtDNA deletions were evaluated in myocardial tissue samples.	3 weeks	Large-scale mtDNA deletion was found to be more likely present in myocardial tissue of paced compared to control animals.

α-MHC: α-myosin heavy chain, ANP: atrial natriuretic peptide, AV: atrioventricular, AVCD: atrioventricular conduction disturbances, BNP: brain natriuretic peptide, CHF: congestive heart failure, CRT: cardiac resynchronization therapy, GAPDH: glyceraldehyde-3-phosphatedehydrogenase, LVEF: left ventricular ejection fraction, LV: left ventricle, mtDNA: mitochondrial DNA, OPA1: optic atrophy 1, RVA: right-ventricular apical pacing, RVOT: right-ventricular outflow tract, SERCA2: sarcoplasmic reticulum calcium ATPase-2, PLN: phospholamban, NPPA: natriuretic peptide A coding gene, NPPB: natriuretic peptide B coding gene, and MYH: myosin heavy chain gene.

**Table 2:** Structural and functional remodeling during RVAP.

Structural Remodeling
Microscopic
Diffuse fibrosis of ECV
Loss of the t-system and separation of RyRs from sarcolemmal structures
Macroscopic
Asymmetrical LV hypertrophy and dilatation
LA dilation
TVR
Functional Remodeling
Reduced stroke volume—Impaired LV twist
Decrease in LV filling duration
fMR
Myocardial coronary perfusion defects

RVAP: Right ventricular apical pacing, ECV: extracellular volume, RyR: ryanodine receptor, LV: left ventricle, LA: left atrium, TVR: tricuspid valve regurgitation, and fMR: functional mitral regurgitation.

## Data Availability

The data that support the findings of this study are openly available.
